# School Belonging and Reading Literacy: A Multilevel Moderated Mediation Model

**DOI:** 10.3389/fpsyg.2022.816128

**Published:** 2022-02-02

**Authors:** Yuting Tan, Zhengcheng Fan, Xiaoman Wei, Tao Yang

**Affiliations:** Collaborative Innovation Center of Assessment Toward Basic Education Quality, Beijing Normal University, Beijing, China

**Keywords:** PISA 2018, school belonging, reading literacy, mastery goal orientation, school disciplinary climate

## Abstract

School belonging is of great significance to students' physical and mental health development, especially academic improvement. However, the mechanism of the influence of school belonging on student academic achievement should be further explored, especially reading performance. Based on ecological systems theory and self-determination theory, the present research constructs a multilevel design to examine a moderated mediation model in which school belonging as a level-1 predictor, mastery goal orientation as a level-1 mediator and school disciplinary climate as a level-2 moderator jointly impact reading literacy. Results of the study were based on the questionnaires from 11,364 (5,455 girls and 5,909 boys) 15-year-olds nested in 332 schools in mainland China that participated in PISA 2018. The cross-sectional analysis indicated that: (1) school belonging had a direct and positive effect on student reading literacy; (2) the relationship between school belonging and reading literacy was prominently mediated by mastery goal orientation; (3) both school disciplinary climate level and strength could negatively moderate the latter half path of “school belonging → mastery goal orientation → reading literacy.” Implications, limitations, and future research directions are discussed.

## Introduction

Academic achievement is an important indicator to evaluate student learning status and the quality of school education. Thus, the study of academic achievement and its influencing factors has always been the general concern of the whole society (Poropat, 2009). Nowadays, increasing researchers focus on the unique role of emotional factors in the cognitive development of students, such as school belonging (Liu and Lu, 2011; Kiefer et al., 2015; Froiland et al., 2016; Okilwa, 2016; Reynolds et al., 2017; Abdollahi and Noltemeyer, 2018; Korpershoek et al., 2019; Dixson, 2020; Huang, 2020; Koyuncu and Firat, 2020; Li et al., 2020). A substantial body of studies conducted in the last decade generally supported the point that school belonging is positively linked with students' learning motivation, mental resilience (Kim and Kim, 2013), growth mindset (Dixson, 2020), and academic success (Korpershoek et al., 2019), while significant negative relations with school belonging and school dropout, absenteeism, violence and, bullying, etc., also were proved (Gini et al., 2018; Korpershoek et al., 2019; Huang, 2020).

Despite prior research literature have established the positive relationship between a student's school belonging and academic achievement (Kiefer et al., 2015; Okilwa, 2016; Reynolds et al., 2017; Abdollahi and Noltemeyer, 2018; Korpershoek et al., 2019; Huang, 2020), several theoretical voids remain. Firstly, domain specificity has been observed in a lot of literature with specific effects of predictors (e.g., gender, socioeconomic status) on different subject domains such as science, reading, and math (Hinnant et al., 2009; Reynolds et al., 2017). However, most existing studies focused on the impact of school belonging on academic achievement that represented a combination of several subjects (Okilwa, 2016; Abdollahi and Noltemeyer, 2018; Dixson, 2020; Li et al., 2020), such as the combination of mathematics, reading, and science (Li et al., 2020) or the combination of mathematics and reading (Okilwa, 2016). Additionally, some studies had also assessed this issue in mathematics (Ho, 2005; Froiland et al., 2016; Reynolds et al., 2017). Thus, there is a need to explore the possible different relations between school belonging and academic outcomes in other disciplinary domains, especially in reading. One reason is that reading literacy is not only a foundation for achievement in other subject areas within the educational system but also a prerequisite for successful participation in most areas of adult life (OECD, 2019; Koyuncu and Firat, 2020). Another is that reading is considered to be a learning experience, which is infused with more emotions compared with other subjects. Nevertheless, little research had been done to focus on the role of emotional factors in reading achievement (Hamedi et al., 2020; Zaccoletti et al., 2020). To fill this gap, the first purpose of this study is to identify the effect of school belonging on student reading performance with a large-scale survey.

Secondly, previous studies have paid more attention to the direct impact of school belonging on academic achievement (Ho, 2005; Liu and Lu, 2011; Okilwa, 2016; Reynolds et al., 2017; Korpershoek et al., 2019; Dixson, 2020; Koyuncu and Firat, 2020), or regarded school belonging as a mediating variable affecting academic outcomes by some variables (Froiland et al., 2016; Huang, 2020; Li et al., 2020), but how and when school belonging affect academic achievement is little known. Several empirical investigations have shown that motivation has been a dominant perspective to explain academic achievement. What's more, reading motivation can better explain the differences in students' reading comprehension than other variables (Rogiers et al., 2020; Ma et al., 2021). Moreover, multiple prior studies supported that school belonging can significantly predict students' learning motivation (e.g., mastery goal orientation) (Neel and Fuligni, 2013; Korpershoek et al., 2019). For instance, a meta-analysis showed that mastery goal orientation, considered to be a proactive and spontaneous learning tendency, was significantly correlated with school belonging, while other kinds of motivations were not (Korpershoek et al., 2019). To the best of the knowledge of the authors, the mediating effect of mastery goal orientation between these two variables has not yet been explored. Thus, another objective of this research is to understand the internal mechanism of this issue, since this could lead to comprehension of how emotional and motivational factors are related to changes in reading achievement.

From the view of constructivism, the specific interaction between learners and environmental factors influences learning outcomes (Chi et al., 2018). For instance, Reynolds et al. (2017) suggested that school climate and school identification (connectedness, belonging, relatedness) are conceptualized as distinct but related concepts and should be investigated in the same model to explain academic development. Speaking of the school environment, one of the key environmental elements is the disciplinary climate (Cheema and Kitsantas, 2014, 2016; Sortkær and Reimer, 2016; Chi et al., 2018), which refers to the degree to which noise and disorder are suppressed, teachers have more time to cover the curriculum and use diverse teaching strategies, and students have time and opportunities to concentrate on academic tasks (Cheema and Kitsantas, 2014, 2016). And existing findings have revealed the moderating effect of disciplinary climate on the relationships between other variables and academic outcomes, such as mathematics performance (Sortkær and Reimer, 2016) and science achievement (Chi et al., 2018), but no similar exploration has been done in the reading context. In this sense, we also need to understand the nature of the interaction between school disciplinary climate and students' internal elements to influence reading literacy. As a result, the extant studies on the link between school belonging and reading literacy will consider the motivational mechanism and climatic boundaries.

Over the past few decades, school belonging has been the most frequently investigated in the United States and other Western countries (Kiefer et al., 2015; Froiland et al., 2016; Okilwa, 2016; Reynolds et al., 2017; Abdollahi and Noltemeyer, 2018; Korpershoek et al., 2019). However, the related research conducted in the Eastern context is still very limited. Considering the differences in cultural and administrative norms between Western and Eastern contexts (Ning et al., 2015), it thus remains unclear whether their theoretical and empirical findings are generalizable to Eastern cultures. For example, in China, cultivating high-performing, motivated, and well-behaved learners now and for life is considered as a combined duty of schooling, which is different from western school systems (Ning, 2020). Thus, we need to continue to study this issue deeper in China to confirm the generalizability of the findings to other cultural and geographic backgrounds. Therefore, this current study aims to provide a deeper understanding of the internal mechanism and conditional boundary underlying the association between student school belonging and reading literacy in a Chinese mainland sample of Programme for International Student Assessment (PISA) 2018.

## Theoretical Foundations and Hypothesis Development

### School Belonging and Reading Literacy

School belonging in educational settings is an important index to evaluate the learning and life quality of students in schools (Huebner, 2004). It has consistently been defined as “the extent to which students feel personally accepted, respected, included, and supported by others in the school social environment” (Goodenow, 1993). According to the self-determination theory (SDT), there are three basic psychological needs of individuals: autonomy, competence, and relationship (Ryan and Deci, 2002), in which relationship needs point out the individual's need for an interpersonal relationship. The sense of belonging is a basic psychological need of individuals (Korpershoek et al., 2019). Consistent with this point, researchers have suggested that students with a higher sense of belonging to school are more likely to show positive psychological outcomes (Korpershoek et al., 2019) and these positive psychological outcomes are associated with academic success (Kiefer et al., 2015; Okilwa, 2016; Reynolds et al., 2017; Abdollahi and Noltemeyer, 2018; Korpershoek et al., 2019), including Chinese students' reading literacy (Huang, 2020).

Although school belonging is often found to be associated with academic achievement, there are some inconsistencies in these findings (Froiland et al., 2016; Dixson, 2020; Koyuncu and Firat, 2020), including Chinese students (Liu and Lu, 2011; Li et al., 2020). For instance, Liu and Lu (2011) found that whether in the initial state or the growth trajectories of the high school transition period, urban public school students' school belonging could non-significantly predict the changes in their academic achievement that was evaluated by the standardized composite scores of Chinese, math, and English. And another study in rural China also reported that school belonging did not predict junior school students' standardized scores of reading, math, and science (Li et al., 2020). As mentioned above, a possible reason for these inconsistent findings in the Chinese samples may be due to the disciplinary differences among reading, math, and science, which were overlooked by the use of combined scores of several subjects. Sample representativeness can also result in this inconsistency. To be specific, a sample was recruited from two urban public high schools located in eastern China (Liu and Lu, 2011) and another sample was represented a rural area of southwestern China (Li et al., 2020), which limit the generalizability of the findings to the whole education situation of the Chinese mainland. Therefore, more empirical research that used a more representative sample should be conducted to get a coherent understanding of the direct effect of school belonging on reading achievement in the Chinese context. Based on SDT and literature analysis, the following assumption was made:

*Hypothesis 1*: School belonging can significantly and positively predict reading literacy.

### Mastery Goal Orientation

Achievement goal orientation theory is considered as one of the most crucial frameworks for understanding learning motivation (Elliot, 1999, 2005), and is often used to predict students' various academic outcomes and comprehend their academic difficulties (Patrick et al., 2011; Was et al., 2017; Diaconu-Gherasim et al., 2019; Zheng et al., 2020). It is generally believed that there are two different patterns: performance goal orientation and mastery goal orientation (Dweek, 1986). Students who demonstrate performance goal orientation believe that learning is to gain high social comparison and external rewards (Elliot, 1999, 2005), while students who tend to mastery goal orientation believe that learning is to acquire new knowledge or skills, gain academic recognition and achieve self-development (Patrick et al., 2011; Was et al., 2017). It can be seen from the above analysis that students with mastery goals generally perform better in academic assessment, and multiple empirical studies also support this point (Shim et al., 2008; King and McInerney, 2016; Diaconu-Gherasim et al., 2019; Korpershoek et al., 2019; Theis et al., 2020).

According to prior studies, students under high levels of school belonging tend to prefer mastery goals, rather than performance goals (Aerts et al., 2015; Korpershoek et al., 2019). And which is demonstrated by SDT, that is external environmental factors influence individual behavior and mental health (e.g., academic achievement) through the mediating effect of internal psychological needs (Jang et al., 2009). Based on SDT and the aforementioned research-derived relationships between school belonging, mastery goal orientation, and academic performance, we found ample evidence that indicated mastery goal orientation could represent a potential mediating role between school belonging and reading literacy. Therefore, the following hypothesis was proposed:

*Hypothesis 2*: Mastery goal orientation mediates the link between school belonging and reading literacy.

### School Disciplinary Climate

Disciplinary climate is conceptualized as the perceptions that students hold on the consistency of classroom rules and how teachers address behavioral problems during class (Cheema and Kitsantas, 2014). And the school disciplinary climate focuses on school discipline and school order from the lens of school members' shared perceptions (Guo et al., 2018). Based on the organizational psychology perspective, climate subsumes two main aspects: climate level and climate strength (Schneider et al., 2013). Climate level refers to the quality of climate, which can be described as positive or negative; climate strength reflects the consensus or agreement on individual perceptions, which can be described as strong or weak (Schneider et al., 2002, 2013, 2017). Accordingly, a positive effect of climate level is usually expected when the climate strength is strong (Schneider et al., 2017).

Ecological systems theory also indicated that the school, as an important environment in the microsystem, has an increasingly strong influence on students (Bronfenbrenner, 1992). From the perspective of evidence, most school disciplinary climate studies have paid more attention to climate level and little focus on climate strength (Guo et al., 2018). These studies mainly supported the positive association between disciplinary climate level and academic behavior (Arum and Velez, 2012; Frempong et al., 2012; Cheema and Kitsantas, 2014; Ning et al., 2015; Jenkins and Ueno, 2017; Chi et al., 2018; Guo et al., 2018; Ning, 2019, 2020), including reading achievement (Ning et al., 2015). Meanwhile, Guo et al. (2018) highlighted the positive effect of disciplinary climate strength on student reading performance. However, to the best knowledge of authors, few studies have identified the impact of school disciplinary climate on students' academic achievement from the lens of both climate level and climate strength. In addition, the moderating effect of disciplinary climate has been found in the relationship between other variables and learning outcomes (Sortkær and Reimer, 2016; Chi et al., 2018). For instance, Sortkær and Reimer (2016) found that the interaction between gender and disciplinary climate significantly influences student mathematics performance, and Chi et al. (2018) showed that disciplinary climate could moderate the association between inquiry-based science activities and student science achievement for both genders. Based on the above findings of other disciplines, the following hypothesis was raised in the reading context:

*Hypothesis 3*: School disciplinary climate level moderates the indirect effect of school belonging and reading literacy through mastery goal orientation.
*Hypothesis 4*: School disciplinary climate strength moderates the indirect effect of school belonging and reading literacy through mastery goal orientation.

**Figure 1:**
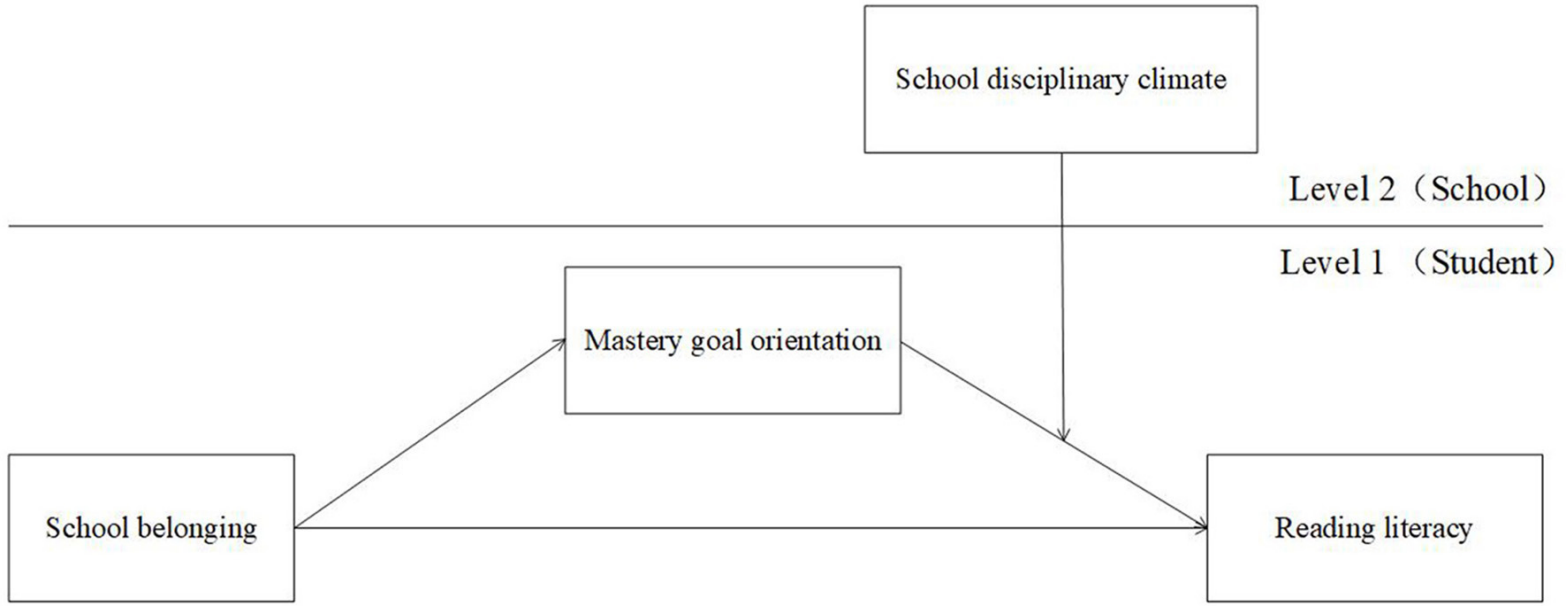
Proposed moderated mediation model.

To sum up, this study constructed a cross-level moderated mediation model based on ecological systems theory and SDT (see Figure 1). The research aimed to explore three main research questions: (1) whether school belonging can directly predict student reading literacy, (2) whether mastery goal orientation has a mediating effect on the relationship between school belonging and reading literacy, and (3) whether the school disciplinary climate has a moderating effect on this mediating model. Testing the above questions would help to clarify the motivational mechanism (i.e., mastery goal orientation) and conditional boundaries (i.e., school disciplinary climate level and strength) underlying the association between school belonging and reading literacy for Chinese mainland students, and provide theoretical basis and empirical support for improving middle school students' reading literacy.

## Materials and Methods

### Participants

PISA is a triennial international survey designed to evaluate education systems worldwide by testing the skills and knowledge of 15-year-old students, together with their background and non-cognitive characteristics and school context. PISA 2018 is the seventh test of the project and also provides multiple reference standards for student evaluation by collecting relevant variables of students' families, teachers, and schools. In the two-stage unequal probability sampling design adopted by PISA, a total of 362 schools were selected from Beijing, Shanghai, Jiangsu, and Zhejiang provinces in China. At each sampled school, as many as 35 students were randomly selected. After preliminary data cleaning and weighting by PISA, 12058 (5,775 girls and 6,283 boys) students that represented 992,302 15-year-old students in mainland China were remained. In addition, considering the requirements of sufficient statistical power (Kreft and de Leeuw, 1998; Kim and Hong, 2020), 30 schools with <30 students were excluded in this study so that each school has a maximum of 35 students and a minimum of 30 students after data cleaning. And the missing data on student-level measures was also deleted for HLM analysis (Enders, 2010), resulting in a total of 11,364 students from 332 schools (5,455 girls and 5,909 boys) were remained.

### Measures

#### Reading Literacy

According to PISA 2018 reading framework, “reading literacy is understanding, using, evaluating, reflecting on and engaging with texts in order to achieve one's goals, to develop one's knowledge and potential and to participate in society (OECD, 2019).” Based on this definition, reading literacy assessment in PISA 2018 was developed by examining three cognitive processes, such as locating information, understanding, evaluating, and reflecting in both simple and multiple texts. Both multiple-choice and open constructed-response items were used. To be clear, participants' reading literacy was assessed in the official language of their home country (here: Chinese). Ten plausible values of reading literacy were displayed in the results of PISA 2018, in which the results were standardized with an average value of 500 and a standard deviation of 100 for OECD countries. In this study, we showed the pooled results that used 10 plausible values for reading literacy to analyze (OECD, 2009).

#### School Belonging

School belonging was represented by the level of acceptance, respect, and support those students feel in their schools (OECD, 2019), the BELONG index in the PISA 2018 student database. This is a standardized index with an average value of zero and a standard deviation of one for the OECD countries. Technically, the BELONG index was calculated by averaging students' levels of agreement with six statements regarding their feelings in their schools, such as “I make friends easily at school.” The response scale ranges from 1 to 4, indicating from “strongly agree” to “strongly disagree,” respectively. In addition, items 1, 4, and 6 were entitled reverse questions. And these items were reversed so that a higher score corresponds to a higher level of school belonging. The Cronbach's raw α for BELONG in China is 0.84 and the McDonald's ω _*h*_ for MASTGOAL in China is 0.84, which indicates an acceptable level of internal consistency.

#### Mastery Goal Orientation

Mastery goal orientation was represented by the tendency to mastery goals that students show in their study and life (OECD, 2019), the MASTGOAL index in the PISA 2018 student database. This is a standardized index with an average value of zero and a standard deviation of one for the OECD countries. Technically, the MASTGOAL index was calculated by averaging students' levels of agreement with three statements regarding their tendency to mastery goals, such as “My goal is to learn as much as possible.” The response scale ranges from 1 to 5, indicating from “not at all true of me” to “extremely true of me,” respectively. All items were positive so that a higher score corresponds to a higher level of mastery goal orientation. The Cronbach's raw α for MASTGOAL in China is 0.72 and the McDonald's ω _*h*_ for MASTGOAL in China is 0.76, which indicates an acceptable level of internal consistency.

#### School Disciplinary Climate

Disciplinary climate is measured by the extent to which students miss learning opportunities due to disruptive behavior in the reading classroom (OECD, 2019). In this study, school disciplinary climate represented the average level of classroom disciplinary climate in a school, with a higher score indicating an orderly climate. The DISCLIMA index was calculated by averaging students' levels of agreement with five statements in their reading lessons, such as “Students don't listen to what the teacher says.” The response scale ranges from 1 to 4, indicating from “every lesson” to “never or hardly ever,” respectively. All items were positive so that higher scores correspond to a better disciplinary climate. The Cronbach's raw α for DISCLIMA in China is 0.89 and the McDonald's ω _*h*_ for DISCLIMA in China is 0.90, which indicates an acceptable level of internal consistency.

In addition to the aforementioned scales, the economic, social, and cultural status index (ESCS) and GENDER which were listed in PISA 2018 database, were used in the analyses. ESCS is a standardized index with an average value of zero and a standard deviation of one for the OECD countries. Three indices were used in the construction of the ESCS index, including the highest occupational status of parents (HISEI), the highest educational level of parents in years of education (PARED), and home possession (HOMEPOS). GENDER was coded as a dummy variable with a value of 1 for girls (*n* = 5,455) and 0 for boys (*n* = 5,909).

### Analytical Strategy

Considering the nested feature of the data (i.e., students in the same school shared the same school disciplinary climate), multilevel modeling (MLM) was employed to analyze the data of the present research. The hypothetical model was tested with the MLmed Beta 2 macro for SPSS software (Hayes and Rockwood, 2020). Utilizing this analytic approach, a multilevel moderated mediation model was proposed. It estimated (see Figure 1) school disciplinary climate as a level-2 moderator of the level-1 indirect effect of school belonging on reading literacy via mastery goal orientation.

Before the formal analysis, the intra-class correlation coefficient (ICC) values of the moderator (i.e., school disciplinary climate) were calculated to estimate the dependence magnitude (Cohen, 1988). The ICC agreements were calculated to examine whether disciplinary climate could significantly explain the variance in individual responses [ICC(1)] and to assess the reliability of school-level means [ICC(2), Bliese, 2000]. R_wg_ was also used to test the polymerization criteria for school disciplinary climate (James et al., 1993). The results showed that ICC(1) = 0.13 (>0.12), ICC(2) = 0.84 (>0.47), and R_wg_ = 0.88 (>0.7), which indicates that a decent proportion of the total variation found in all student ratings can be attributed to the fact that students are nested within schools.

Consistent with previous research, the school mean of students' responses on disciplinary climate is used as an index for disciplinary climate level (Chan, 1998), and within-school standard deviation of disciplinary climate scores is used to indicate climate strength (Roberson et al., 2007). Given that the standard deviation is an index of variability, it is multiplied by −1, so that a higher score represents a higher climate strength (Guo et al., 2018).

## Results

### Confirmatory Factor Analysis

We use 14 response items measuring the three constructs (six items of school belonging, three items of mastery goal orientation, and five items of school disciplinary climate) to test the CFA in Mplus 8.0 to merge the variables gradually and examine changes in fitting degrees to test the discriminant validity of the model. The fitting indices of the one-factor model were not good: χ^2^*/df* = 434.03, *CFI* = 0.51, *TLI* = 0.42, *RMSEA* = 0.20 and *SRMR* = 0.17. Secondly, we combined school belonging and mastery goal orientation, and the fitting indices of the two-factor model were still not satisfactory: χ^2^*/df* = 172.84, *CFI* = 0.81, *TLI* = 0.77, *RMSEA* = 0.12 and *SRMR* = 0.08. At last, the result of three-factor model indicated a good fit to the data: χ^2^*/df* = 8.31, *CFI* = 0.99, *TLI* = 0.99, *RMSEA* = 0.03 and *SRMR* = 0.02. And the loading of each item in the three-factor model were shown in Table 1.

**Table 1:** The loading of each item.

**Scale**	**Items' number & loading**					
Mastery goal orientation	1	2	3			
	0.51	0.82	0.83			
School disciplinary climate	1	2	3	4	5	
	0.72	0.71	0.72	0.88	0.86	
School belonging	1	2	3	4	5	6
	0.79	0.57	0.86	0.69	0.54	0.79

Then, Harman's One-Factor Test was conducted to examine the common method biases. And the result reported that the first factor accounted for 31.54% (<40%) of the total variance, which indicated that the influence of the homologous coefficient of variance was not serious.

### Descriptive Statistical Analysis

In this study, both disciplinary climate level and disciplinary climate strength were at the school level (i.e., level-2), whereas school belonging, mastery goal orientation, and reading literacy were at the student level (i.e., level-1). The control variables (i.e., gender and ESCS) were included in the model as student-level variables. Descriptive statistics and correlations between the variables are shown in Table 2. The significant coefficients of pairwise correlation range between −0.03 and 0.54.

**Table 2:** Descriptive statistics and correlation coefficient (*n* = 11,364).

	**M**	**SD**	**1**	**2**	**3**	**4**	**5**	**6**
1 Gender	0.48	0.50	-					
2 ESCS	−0.34	1.07	0.02^*^	-				
3 School belonging	−0.15	0.91	−0.03^**^	0.14^***^	-			
4 Mastery goal orientation	0.06	0.91	0.02^**^	0.16^***^	0.26^***^	-		
5 School disciplinary climate level	0.82	0.42	0.05^***^	0.29^***^	0.14^***^	0.14^***^	-	
6 School disciplinary climate strength	−0.94	0.17	0.04^***^	0.10^***^	0.03^***^	0.04^***^	0.54^***^	
7 Reading literacy	564.58	88.26	0.08^***^	0.36^***^	0.07^***^	0.14^***^	0.38^***^	0.27^***^

* *p < 0.05*,

** *p < 0.01*,

*** *p < 0.001*.

Before testing the hypotheses, we examined the variations in reading literacy across the level. The result shows that level-1 variance was 53.64% of the total variance, and level-2 variance was 46.36% of the total variance. These results indicated a significant variance at level-2 for reading literacy (James, 1982). Thus, using a multilevel model was appropriate.

### Hypothesis Testing

**Figure 2:**
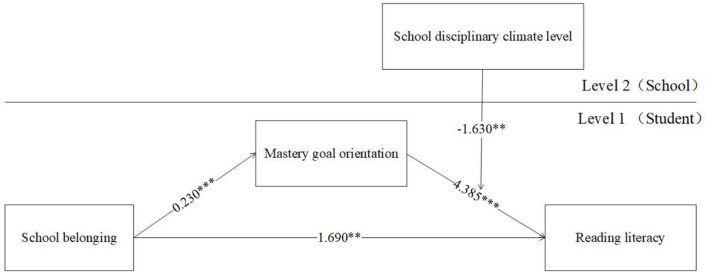
Moderated mediation analysis for Hypothesis 3.

**Figure 3:**
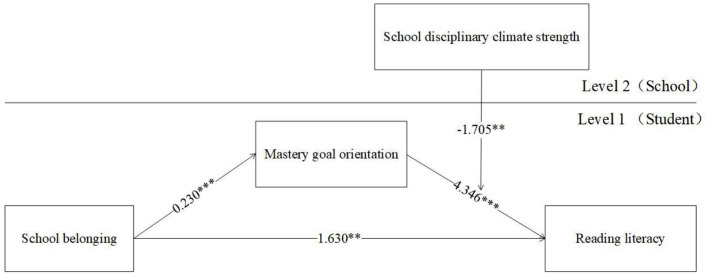
Moderated mediation analysis for Hypothesis 4.

MLM was employed to verify the hypothesis of the present research. There are two models to be tested because there are two moderators (i.e., school disciplinary climate level and school disciplinary climate strength). The results of the moderated mediation analysis are reported in Table 3 and Figures 2, 3. As seen in Figures 2, 3, no matter which moderator was considered, the direct effect of school belonging on reading literacy at the student level was always significant and positive [effect = 1.690, 95% CI (0.397, 2.983); effect = 1.630, 95% CI (0.336, 2.923)], hence hypotheses 1 was supported.

**Table 3:** Indirect effect and moderated mediation effect.

	**School disciplinary climate level**				**School disciplinary climate strength**			
	**Effect**	**SE**	**LL**	**UL**	**Effect**	**SE**	**LL**	**UL**
Indirect effect	1.008	0.157	0.694	1.320	0.999	0.157	0.684	1.311
Index of moderated mediation	−0.374		−0.660	−0.096	−0.392		−0.682	−0.108
Low (-1SD)	1.382	0.209	0.972	1.807	1.390	0.209	0.980	1.877
High (+1SD)	0.633	0.216	0.202	1.057	0.607	0.220	0.168	1.038

Hypothesis 2 tested the mediating effect of mastery goal orientation on the link between school belonging and reading literacy. As shown in Table 3, no matter which moderator was considered, the indirect effect of school belonging on reading literacy via mastery goal orientation at the student level was always significant and positive [effect = 1.008, 95% CI (0.694, 1.320); effect = 0.999, 95% CI (0.684, 1.311)], so that hypotheses 2 was verified.

**Figure 4:**
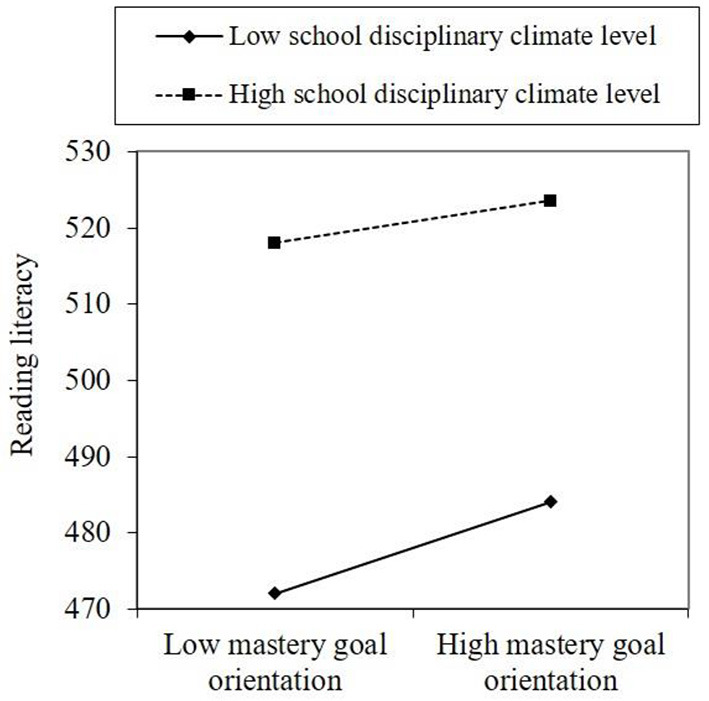
Moderated mediation plot of Hypothesis 3.

**Figure 5:**
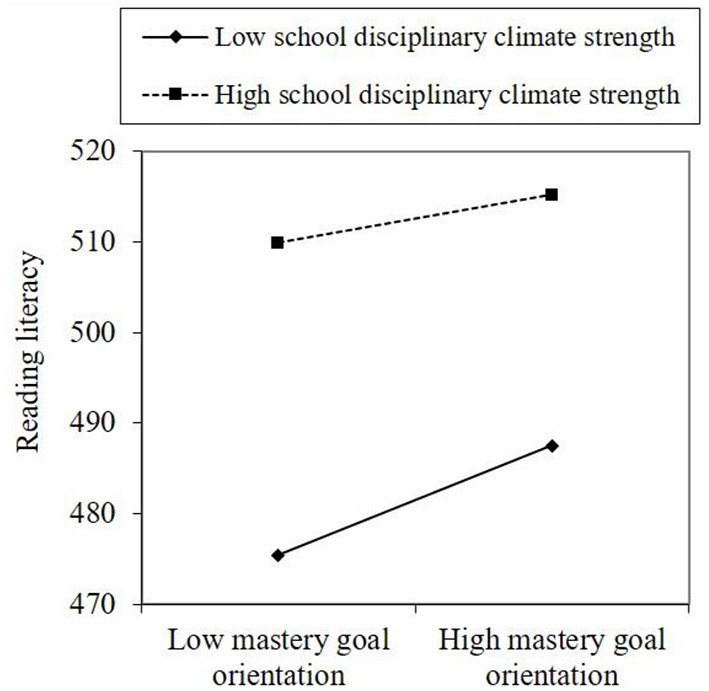
Moderated mediation plot of Hypothesis 4.

Hypothesis 3 and 4 tested the moderated roles of school disciplinary climate level and strength on the indirect effect of school belonging and reading literacy through mastery goal orientation. Figure 2 shows that a significant and negative interaction effect was found in school disciplinary climate level moderated the link between mastery goal orientation and reading literacy [*b* = −1.630, 95% CI (−2.759, −0.410)]. Similarly, Figure 3 also shows that a significant and negative interaction effect was observed in which school disciplinary climate strength moderated the link between these two variables [*b* = −1.705, 95% CI (−2.945, −0.464)]. The results in Table 3 show that both indexes of moderated mediation were significant and negative [effect = −0.374, 95% CI (−0.660, −0.096); effect = −0.392, 95% CI (−0.682, −0.108)]. The conditional indirect effect of school belonging on reading literacy at a low level of school disciplinary climate level [effect = 1.382, 95% CI (0.972, 1.807)] was higher than the effect at a high level of school disciplinary climate level [effect =0.633, 95% CI (0.202, 1.057)], Hypotheses 3 was proved (see Table 3 and Figure 4). Similarly, the conditional indirect effect of school belonging on reading literacy at a low level of school disciplinary climate strength [effect = 1.390, 95% CI (0.980, 1.877)] was higher than the effect at a high level of school disciplinary climate strength [effect =0.607, 95% CI (0.168, 1.038)], Hypotheses 4 was proved (see Table 3 and Figure 5).

## Discussion

How to improve students' academic achievement is an essential consideration for all school educators and policymakers, as well as students' well-being and non-cognitive development. Based on ecological systems theory and SDT, the present research provides valuable insights into how school belonging affects Chinese mainland students' reading literacy and also gives consideration to the impact of mastery goal orientation as a critical mediator. Furthermore, we also reveal the interplay between school disciplinary climate (i.e., climate level and climate strength) and mastery goal orientation influencing students' reading literacy by performing a multilevel moderated mediation analysis. And this present study contributes to the existing literature as outlined below.

In the first place, the positive direct effect of school belonging on reading literacy was clarified and confirmed in the Chinese context. This finding was in line with theoretical expectations, most previous works that were conducted in Western counties (Kiefer et al., 2015; Okilwa, 2016; Reynolds et al., 2017; Abdollahi and Noltemeyer, 2018; Korpershoek et al., 2019) and one study that especially centered on Chinese students' reading literacy in PISA (Huang, 2020), but was inconsistent with some results that focused on a small sample of Chinese students, whose academic achievement represented a combination of three subjects (Liu and Lu, 2011; Li et al., 2020). Draw on the above analysis, both discipline differences (Hinnant et al., 2009; Reynolds et al., 2017) and sample representativeness could lead to the inconsistency in these studies. In consistent with SDT (Ryan and Deci, 2002), one explanation for the positive relationship may lie in the higher level of school belonging provided for students by respect and care from teachers, peers, and schools, which meet students' basic psychological needs, heighten their motivation (Neel and Fuligni, 2013), self-efficacy (Dixson, 2020), and other internal psychological factors (Korpershoek et al., 2019), and even hinder the influence of negative factors such as academic pressure and anxiety (Abdollahi et al., 2020), as a result, it can improve academic outcomes. Once again, the result of the current study affirms that valid and abundant emotional interactions with classmates and teachers can not only enhance adolescents' reading comprehension and performance, but also promote their cognitive development and academic prospects.

Regarding the second hypothesis, we empirically support the idea that mastery goal orientation partially mediates the positive relationship between school belonging and reading literacy, serving as a more powerful explanatory mechanism. In consistent with SDT, school belonging has the potential to encourage students and to transform their emotional energy into better mastery goals and then advanced reading literacy. It seems reasonable to assume that positive relationships with classmates and teachers, which imply a high sense of belonging to school, could increase more positive emotions such as hope, happiness, and pride and less negative emotions such as boredom and anger (Pekrun et al., 2006, 2009), and strengthen their perception of mastery goals (Patrick et al., 2011). In addition, a plethora of studies noted that mastery goal orientation can effectively facilitate students' metacognitive strategies (King and McInerney, 2016), working memory (Wolters, 2004) and classroom participation (Datu and Park, 2019; Yi et al., 2020), self-management (Skinner et al., 2009) and other positive variables associated with academic achievement, can also resist negative factors such as academic pressure and anxiety (Zheng et al., 2020), so that can improve academic achievement (Wolters, 2004; King and McInerney, 2016; Diaconu-Gherasim et al., 2019; Theis et al., 2020). Therefore, this result may explain the nonsignificant direct effect of school belonging on Chinese students' academic achievement (Liu and Lu, 2011; Li et al., 2020). It is not that the sense of belonging to school does not directly predict academic development, but indirectly through other variables such as mastery goal orientation. In summary, this finding contributes to the literature by verifying a theoretical model of the mechanism between individual emotional characteristics and motivational factors in achieving academic success.

At last, perhaps the most important and novel finding of this study was that both school disciplinary climate level and climate strength could moderate the link between mastery goal orientation and reading literacy, and the indexes of moderated mediation were all significant and negative. This result was consistent with the neural findings, for example, Braver et al. (2014) have reported that an orderly school disciplinary climate is extremely crucial in the motivation-achievement relationship for adolescents, considering that most adolescents' prefrontal cortex, which is in charge of self-control, has not fully matured. And in line with the ecological systems theory, this study admitted the importance of the interaction between the learning environment (i.e., school disciplinary climate) and student internal characteristics (i.e., mastery goal orientation) for academic promotion. Conceptually, in a strong climate, explicit expectations can lead people to act similarly. While people behave differently in a weak climate, the organizational goals and values are too obscure for them to form consistent perceptions (Schneider et al., 2002, 2013, 2017). Especially, only when students' perception of school disciplinary climate is both positive and consistent, can the school disciplinary climate be acceptable for school members (Guo et al., 2018). In other words, there is a significant correlation between the level and strength of school disciplinary climate. Therefore, these two similar moderated mediation indexes are understandable and logical.

To be specific, the conditional indirect effect of school belonging on reading literacy at a low level of school disciplinary climate was greater than the effect at a high level of school disciplinary climate. Further analysis revealed that the reason is that the impact of the mastery goal orientation on student reading literacy can be weakened by both a more positive school disciplinary climate level and strong climate strength, but be enhanced by both a more negative school disciplinary climate level and weak climate strength. On the one hand, it is possible that a highly ordered school disciplinary climate would undermine student sense of self-determination, intrinsic motivation (Kover and Worrell, 2010), and autonomy (Ning, 2020), especially in East Asian cultures, where an orderly learning atmosphere and explicit rules are established to hinder distraction, noise, and disorder in the classroom (Ning, 2019, 2020), which could easily lead to the weakening of students' tendency to mastery goals. On the other hand, although the negative and weak school disciplinary climate always means loose school management, students under higher self-management ability and autonomy are more likely to tend to mastery goals and then take the initiative to utilize abundant resources to make academic progress. This may explain why both higher school disciplinary climate level and strength would weaken the relationship between mastery goal orientation and reading literacy, while a strong link between these two variables was observed at both low disciplinary climate level and strength. On the basis of this finding, the importance of interaction between individual motivational factors and external environment is highlighted in achieving academic success in the Chinese context. However, research in this area is limited and further works are urgently needed to investigate the potential boundary conditions of the effect of school belonging on learning outcomes to understand the changes in reading behavior.

And these results enrich the research on the relationship between school belonging and academic achievement and provide a practical basis for policymakers, school administrators, and teachers to improve the reading literacy of students and entire educational quality. In the first place, the empirical results support the valuable idea that school belonging is significant for students' development. Therefore, it is urgent to take effective measures to help students who may have such problems develop a sense of belonging to school. Secondly, students with mastery goal orientation are more likely to channel positive and powerful emotional energy into academic achievement. Hence, the educational significance of this finding is emphasized by the fact that mastery goal orientation is a factor that is relatively more manipulable compared to some other vital predictors of academic success such as socioeconomic status. However, mastery goal orientation seems to pose a differential impact on reading literacy depending on the different levels of disciplinary climate level and strength. The pursuit of an orderly disciplinary climate would regulate the bad behavior of students and provide a powerful safeguard for teaching and learning, but may impair the initiative and autonomy of students with mastery goals, and even weaken the link between positive psychological factors (i.e., mastery goal orientation) and academic achievement. Certainly, we need more relevant research to assist policymakers, school administrators, and teachers to address the issue of creating a more temperate disciplinary environment.

### Limitations and Implications for Future Research

Three limitations of the present research should be noted to provide directions for future research. Firstly, we used the data from PISA 2018 that was published by OECD. These results represent the education situation of 15-year-old students from Beijing, Shanghai, Jiangsu, and Zhejiang provinces in China, which does not necessarily represent the education status of the whole country. In addition, because the definition and optimal level and strength of a school discipline climate may vary from culture to culture, the successful findings and practices on school disciplinary climate found in one country may not be suitable for the schools in other countries (Guo et al., 2018). Therefore, we need more effort in other subjects and cultures. Secondly, in the cross-sectional design of PISA 2018, it is limited to make directional assumptions only from the data in this study. Thus, we cannot fully reveal the causal relationship between these variables, so that we urge future research to replicate our findings using multiple sources and multiple time points in data collection. Finally, PISA examined a wide range of variables in their student self-reported questionnaire, which is prone to common method deviation. Although we have proved that the possible common method deviation is not serious and the tools have higher reliability and validity according to the statistical results, such situations should still be avoided in future studies. Future research could overcome this limitation by considering objective methods (e.g., observations and interviews) to evaluate the relationships between variables.

## Conclusion

According to ecological systems theory and SDT, from both motivational and environmental point of view, the present research establishes a cross-level model to explain how and when school belonging impact students' reading literacy in the Chinese context. After performing the cross-sectional analysis, the results indicated that: (1) school belonging had a direct and positive effect on student reading literacy; (2) the relationship between school belonging and reading literacy was prominently mediated by mastery goal orientation; (3) both school disciplinary climate level and strength could negatively moderate the latter half path of “school belonging → mastery goal orientation → reading literacy.” Notwithstanding the preliminary nature of our findings, we have some confidence in these conclusions as they are based on a sample from a large-scale survey in the Chinese context, affording a high degree of external validity. Nevertheless, there is still much work that remains to be done in this emerging line of research and future work needs to consolidate and extend our findings.

## Data Availability Statement

Publicly available datasets were analyzed in this study. This data can be found at: https://www.oecd.org/pisa/data/2018database.

## Author Contributions

YT and TY designed this study. YT analyzed the data and wrote this article. ZF, XW, and TY reviewed the study and performed substantial suggestions. All authors contributed to the article and approved the submitted version.

## Funding

This study was supported by the Independent project of Collaborative Innovation Center of Assessment toward Basic Education Quality (China) (BJZK-2020A1-20007).

## Conflict of Interest

The authors declare that the research was conducted in the absence of any commercial or financial relationships that could be construed as a potential conflict of interest.

## Publisher's Note

All claims expressed in this article are solely those of the authors and do not necessarily represent those of their affiliated organizations, or those of the publisher, the editors and the reviewers. Any product that may be evaluated in this article, or claim that may be made by its manufacturer, is not guaranteed or endorsed by the publisher.

## References

[B1] Abdollahi A. Noltemeyer A. (2018). Academic hardiness: Mediator between sense of belonging to school and academic achievement? J. Educ. Res. 111, 345–351. 10.1080/00220671.2016.1261075

[B2] Abdollahi A. Panahipour S. Tafti M. A. Allen K. A. (2020). Academic hardiness as a mediator for the relationship between school belonging and academic stress. Psychol. Sch. 57, 823–832. 10.1002/pits.2233925855820

[B3] Aerts S. Houtte M. V. Dewaele A. Cox N. Vincke J. (2015). School motivation in secondary schools: A survey of LGB and heterosexual students in Flanders. Youth Soc. 47, 412–437. 10.1177/0044118X12467657 22269049

[B4] Arum R. Velez M. (2012). Improving Learning Environments: School Discipline and Student Achievement in Comparative Perspective (1st ed.). Stanford, CA: Stanford University Press. 10.11126/stanford/9780804778039.003.0001

[B5] Bliese P. D. (2000). Within-group agreement, non-independence, and reliability: Implications for data aggregation and analysis, in Multilevel Theory, Research, and Methods in Organizations, eds Klein K. J. Kozlowski S. W. J. (San Francisco, CA: Jossey-Bass).

[B6] Braver T. S. Krug M. K. Chiew K. S. Kool W. Westbrook J. A. Clement N. J.. (2014). Mechanisms of motivation-cognition interaction: Challenges and opportunities. Cogn. Affect. Behav. Neurosci. 14, 443–472. 10.3758/s13415-014-0300-0 24920442 PMC4986920

[B7] Bronfenbrenner U. (1992). Ecological systems theory, in Six Theories of Child Development: Revised Formulations and Current Issues, ed Vasta R. (London: Jessica Kingsley Publishers).

[B8] Chan D. (1998). Functional relations among constructs in the same content domain at different levels of analysis: A typology of composition models. J. Appl. Psychol. 83, 234–246. 10.1037/0021-9010.83.2.234

[B9] Cheema J. R. Kitsantas A. (2014). Influences of disciplinary classroom climate on high school student self-efficacy and mathematics achievement: A look at gender and racial-ethnic differences. Int. J. Sci. Mathematics Educ. 12, 1261–1279. 10.1007/s10763-013-9454-4

[B10] Cheema J. R. Kitsantas A. (2016). Predicting high school student use of learning strategies: The role of preferred learning styles and classroom climate. Educ. Psychol. 36, 845–862. 10.1080/01443410.2014.981511

[B11] Chi S. Liu X. Wang Z. Han S. W. (2018). Moderation of the effects of scientific inquiry activities on low SES students' PISA 2015 science achievement by school teacher support and disciplinary climate in science classroom across gender. Int. J. Sci. Educ. 40, 1284–1304. 10.1080/09500693.2018.1476742

[B12] Cohen J. (1988). Statistical Power Analysis for the Behavioral Sciences (2nd ed.). New York, NY: Routledge.

[B13] Datu J. A. D. Park N. (2019). Perceived school kindness and academic engagement: The mediational roles of achievement goal orientations. School Psychol. Int. 40, 456–473. 10.1177/0143034319854474

[B14] Diaconu-Gherasim L. R. Tepordei A.-M. Mairean C. Rusu A. (2019). Intelligence beliefs, goal orientations and children's academic achievement: Does the children's gender matter? Educ. Stud. 45, 95–112. 10.1080/03055698.2018.1443796

[B15] Dixson D. D. (2020). How hope measures up: Hope predicts school variables beyond growth mindset and school belonging. Curr. Psychol. 10.1007/s12144-020-00975-y

[B16] Dweek C. S. (1986). Motivational processes affecting learning. Am. Psychol. 41, 1040–1048. 10.1037/0003-066X.41.10.1040

[B17] Elliot A. J. (1999). Approach and avoidance motivation and achievement goals. Educ. Psychol. 34, 169–189. 10.1207/s15326985ep3403_3

[B18] Elliot A. J. (2005). A conceptual history of the achievement goal construct, in Handbook of Competence and Motivation, eds Elliot A. J. Dweek C. S. (New York, NY: Guilford Publications).

[B19] Enders C. K. (2010). Applied Missing Data Analysis (Illustrated edition). New York, NY: The Guilford Press.

[B20] Frempong G. Ma X. Mensah J. (2012). Access to postsecondary education: can schools compensate for socioeconomic disadvantage? Higher Educ. 63, 19–32. 10.1007/s10734-011-9422-2

[B21] Froiland J. M. Davison M. L. Worrell F. C. (2016). Aloha teachers: Teacher autonomy support promotes Native Hawaiian and Pacific Islander students' motivation, school belonging, course-taking and math achievement. Soc. Psychol. Educ. 19, 879–894. 10.1007/s11218-016-9355-9

[B22] Gini G. Marino C. Pozzoli T. Holt M. (2018). Associations between peer victimization, perceived teacher unfairness, and adolescents' adjustment and well-being. J. Sch. Psychol. 67, 56–68. 10.1016/j.jsp.2017.09.005 29571535

[B23] Goodenow C. (1993). The psychological sense of school membership among adolescents: Scale development and educational correlates. Psychol. Sch. 30, 79–90. 10.1002/1520-6807(199301)30:1<79::AID-PITS2310300113>3.0.CO

[B24] Guo S. Li L. Zhang D. (2018). A multilevel analysis of the effects of disciplinary climate strength on student reading performance. Asia Pacific Educ. Rev. 19, 1–15. 10.1007/s12564-018-9516-y

[B25] Hamedi S. M. Pishghadam R. Fadardi J. S. (2020). The contribution of reading emotions to reading comprehension: The mediating effect of reading engagement using a structural equation modeling approach. Educ. Res. Policy Pract. 19, 211–238. 10.1007/s10671-019-09256-3

[B26] Hayes A. F. Rockwood N. J. (2020). Conditional process analysis: Concepts, computation, and advances in the modeling of the contingencies of mechanisms. Am. Behav. Sci. 64, 19–54. 10.1177/0002764219859633

[B27] Hinnant J. B. O'Brien M. Ghazarian S. R. (2009). The longitudinal relations of teacher expectations to achievement in the early school years. J. Educ. Psychol. 101, 662–670. 10.1037/a0014306 20428465 PMC2860190

[B28] Ho E. S. C. (2005). Effect of school decentralization and school climate on student mathematics performance: The case of Hong Kong. Educ. Res. Policy Practice 4, 47–64. 10.1007/s10671-005-1546-7

[B29] Huang L. (2020). Exploring the relationship between school bullying and academic performance: The mediating role of students' sense of belonging at school. Educ. Stud. 10.1080/03055698.2020.1749032

[B30] Huebner E. S. (2004). Research on assessment of life satisfaction of children and adolescents. Soc. Indic. Res. 66, 3–33. 10.1023/B:SOCI.0000007497.57754.e3

[B31] James L. R. (1982). Aggregation bias in estimates of perceptual agreement. J. Appl. Psychol. 67, 219–229. 10.1037/0021-9010.67.2.219

[B32] James L. R. Demaree R. G. Wolf G. (1993). rwg: An assessment of within-group interrater agreement. J. Appl. Psychol. 78, 306–309. 10.1037/0021-9010.78.2.306

[B33] Jang H. Reeve J. Ryan R. M. Kim A. (2009). Can self-determination theory explain what underlies the productive, satisfying learning experiences of collectivistically oriented Korean students? J. Educ. Psychol. 101, 644–661. 10.1037/a0014241

[B34] Jenkins A. Ueno A. (2017). Classroom disciplinary climate in secondary schools in England: What is the real picture? Br. Educ. Res. J. 43, 124–150. 10.1002/berj.325525855820

[B35] Kiefer S. M. Alley K. M. Ellerbrock C. R. (2015). Teacher and peer support for young adolescents' motivation, engagement, and school belonging. RMLE Online 38, 1–18. 10.1080/19404476.2015.11641184

[B36] Kim D. H. Kim J. H. (2013). Social relations and school life satisfaction in South Korea. Soc. Indic. Res. 112, 105–127. 10.1007/s11205-012-0042-8

[B37] Kim S. Hong S. (2020). Comparing methods for multilevel moderated mediation: a decomposed-first strategy. Struct. Eq. Model. Multidisciplinary J. 25, 661–677. 10.1080/10705511.2019.1683015

[B38] King R. B. McInerney D. M. (2016). Do goals lead to outcomes or can it be the other way around?: causal ordering of mastery goals, metacognitive strategies, and achievement. Br. J. Educ. Psychol. 86, 296–312. 10.1111/bjep.12107 26924161

[B39] Korpershoek H. Canrinus E. T. Fokkens-Bruinsma M. Boer H. de. (2019). The relationships between school belonging and students' motivational, social-emotional, behavioural, and academic outcomes in secondary education: A meta-analytic review. Res. Papers Educ. 35, 641–680. 10.1080/02671522.2019.1615116

[B40] Kover D. J. Worrell F. C. (2010). The influence of instrumentality beliefs on intrinsic motivation: a study of high-achieving adolescents. J. Adv. Acad. 21, 470–498. 10.1177/1932202X1002100305

[B41] Koyuncu I. Firat T. (2020). Investigating reading literacy in PISA 2018 assessment. Int. Elect. J. Element. Educ. 13, 263–275. 10.26822/iejee.2021.189

[B42] Kreft I. G. de Leeuw J. (1998). Introducing Multilevel Modeling. London: Sage. 10.4135/9781849209366

[B43] Li L. Chen X. Li H. (2020). Bullying victimization, school belonging, academic engagement and achievement in adolescents in rural China: A serial mediation model. Child. Youth Serv. Rev. 113:104946. 10.1016/j.childyouth.2020.104946

[B44] Liu Y. Lu Z. (2011). Trajectories of Chinese students' sense of school belonging and academic achievement over the high school transition period. Learn. Individ. Differ. 21, 187–190. 10.1016/j.lindif.2010.12.007

[B45] Ma L. Luo H. Xiao L. (2021). Perceived teacher support, self-concept, enjoyment and achievement in reading: A multilevel mediation model based on PISA 2018. Learn. Individ. Differ. 85:101947. 10.1016/j.lindif.2020.101947

[B46] Neel C. G.-O. Fuligni A. (2013). A longitudinal study of school belonging and academic motivation across high school. Child Dev. 84, 678–692. 10.1111/j.1467-8624.2012.01862.x 23002809

[B47] Ning B. (2019). Examining the importance of discipline in Chinese schooling: An exploration in Shanghai, Hong Kong, Macao, and Taipei. Asia Pacific Educ. Rev. 20, 489–501. 10.1007/s12564-018-9563-4

[B48] Ning B. (2020). Discipline, motivation, and achievement in mathematics learning: An exploration in Shanghai. Sch. Psychol. Int. 41, 595–611. 10.1177/0143034320961465

[B49] Ning B. Damme J. V. Noortgate W. V. D. Yang X. Gielen S. (2015). The influence of classroom disciplinary climate of schools on reading achievement: A cross-country comparative study. School Effect. School Improve. 26, 586–611. 10.1080/09243453.2015.1025796

[B50] OECD (2009). PISA Data Analysis Manual: SPSS (2nd ed). OECD Publishing.

[B51] OECD (2019). PISA 2018 Assessment and Analytical Framework. OECD Publishing. 10.1787/b25efab8-en

[B52] Okilwa N. S. A. (2016). Exploring school- and home-related protective factors for economically disadvantaged middle school students. J. At-Risk Issues 19, 34–46.

[B53] Patrick H. Kaplan A. Ryan A. (2011). Positive classroom motivational environments: Convergence between mastery goal structure and classroom social climate. J. Educ. Psychol. 103, 367–382. 10.1037/a0023311

[B54] Pekrun R. Elliot A. J. Maier M. A. (2006). Achievement goals and discrete achievement emotions: A theoretical model and prospective test. J. Educ. Psychol. 98, 583–597. 10.1037/0022-0663.98.3.583

[B55] Pekrun R. Elliot A. J. Maier M. A. (2009). Achievement goals and achievement emotions: Testing a model of their joint relations with academic performance. J. Educ. Psychol. 101, 115–135. 10.1037/a0013383

[B56] Poropat A. E. (2009). A meta-analysis of the five-factor model of personality and academic performance. Psychol. Bull. 135, 322–338. 10.1037/a0014996 19254083

[B57] Reynolds K. J. Lee E. Turner I. Bromhead D. Subasic E. (2017). How does school climate impact academic achievement? An examination of social identity processes. School Psychol. Int. 38, 78–97. 10.1177/0143034316682295

[B58] Roberson Q. M. Sturman M. C. Simons T. L. (2007). Does the measure of dispersion matter in multilevel research? A Comparison of the relative performance of dispersion indexes. Organizational Res. Methods 10, 564–588. 10.1177/1094428106294746

[B59] Rogiers A. Van Keer H. Merchie E. (2020). The profile of the skilled reader: An investigation into the role of reading enjoyment and student characteristics. Int. J. Educ. Res. 99:101512. 10.1016/j.ijer.2019.101512

[B60] Ryan R. M. Deci E. L. (2002). An overview of self-determination theory: An organismic-dialectical perspective, in Handbook of Self-Determination Research, eds Deci E. L. Ryan R. M. (The University of Rochester Press).

[B61] Schneider B. Ehrhart M. G. Macey W. H. (2013). Organizational climate and culture. Annu. Rev. Psychol. 64, 361–388. 10.1146/annurev-psych-113011-143809 22856467

[B62] Schneider B. González-Romá V. Ostroff C. West M. A. (2017). Organizational climate and culture: Reflections on the history of the constructs in the Journal of Applied Psychology. J. Appl. Psychol. 102, 468–482. 10.1037/apl0000090 28125256

[B63] Schneider B. Salvaggio A. N. Subirats M. (2002). Climate strength: A new direction for climate research. J. Appl. Psychol. 87, 220–229. 10.1037/0021-9010.87.2.220 12002951

[B64] Shim S. S. Ryan A. M. Anderson C. J. (2008). Achievement goals and achievement during early adolescence: Examining time-varying predictor and outcome variables in growth-curve analysis. J. Educ. Psychol. 100, 655–671. 10.1037/0022-0663.100.3.655

[B65] Skinner E. A. Kindermann T. A. Connell J. P. Wellborn J. G. (2009). Engagement and disaffection as organizational constructs in the dynamics of motivational development, in Handbook of Motivation at School, eds Wenzel K. R. Wigfield A. (Portland, OR: Routledge/Taylor & Francis Group).

[B66] Sortkær B. Reimer D. (2016). Disciplinary Climate and Student Achievement: Evidence From Schools and Classrooms. AU Library Scholarly Publishing Services. 10.7146/aul.154.126

[B67] Theis D. Sauerwein M. Fischer N. (2020). Perceived quality of instruction: The relationship among indicators of students' basic needs, mastery goals, and academic achievement. Br. J. Educ. Psychol. 90, 176–192. 10.1111/bjep.12313 31562646

[B68] Was C. A. Al-Harthy I. Stack-Oden M. Isaacson R. M. (2017). Academic identity status and the relationship to achievement goal orientation. Elect. J. Res. Educ. Psychol. 7, 627–652. 10.25115/ejrep.v7i18.136320495610

[B69] Wolters C. A. (2004). Advancing achievement goal theory: Using goal structures and goal orientations to predict students' motivation, cognition, and achievement. J. Educ. Psychol. 96, 236–250. 10.1037/0022-0663.96.2.236

[B70] Yi H. Tian L. Huebner E. S. (2020). Mastery goal orientations and subjective well-being in school among elementary school students: The mediating role of school engagement. Eur. J. Psychol. Educ. 35, 429–450. 10.1007/s10212-019-00431-x

[B71] Zaccoletti S. Altoe G. Mason L. (2020). The interplay of reading-related emotions and updating in reading comprehension performance. Br. J. Educ. Psychol. 90, 663–682. 10.1111/bjep.12324 31654408

[B72] Zheng J. Jiang N. Dou J. (2020). Autonomy support and academic stress: A relationship mediated by self-regulated learning and mastery goal orientation. New Waves-Educ. Res. Dev. J. 23, 43–63.

